# Supplementary material for: The Mi-2 nucleosome remodeler and the Rpd3 histone deacetylase are involved in piRNA-guided heterochromatin formation
Source: Nat Commun. 2020 Jun 4;11:2818. doi: 10.1038/s41467-020-16635-5 (PMC7272611; doi:10.1038/s41467-020-16635-5)
Supplement: Supplementary file 1 — Supplementary Information [file 41467_2020_16635_MOESM1_ESM.pdf]

SUPPLEMENTARY INFORMATION

The Mi-2 nucleosome remodeler and the Rpd3 histone deacetylase are involved in piRNA-guided heterochromatin formation

Mugat et al.

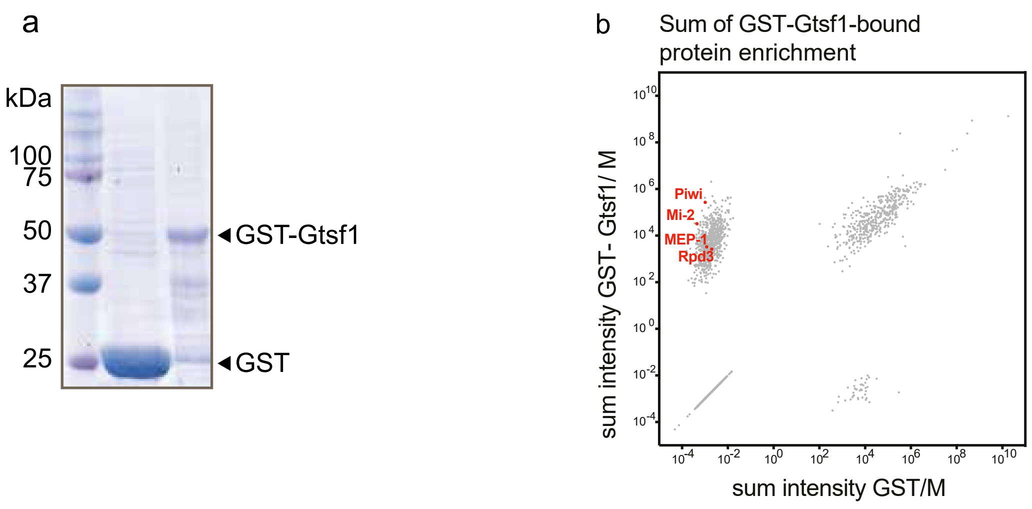

Supplementary Figure 1: GST pull-down of OSC nuclear extracts with recombinant GST-Gtsf1 fusion protein.

a) Coomassie blue staining of the gel with purified GST and GST-Gtsf1 proteins. b) Scatter plot showing fold enrichment of proteins determined by mass spectrometry in GST-Gtsf1 pull-down versus GST control. Proteins of interest are indicated in red. (Biological replicate: n=1).

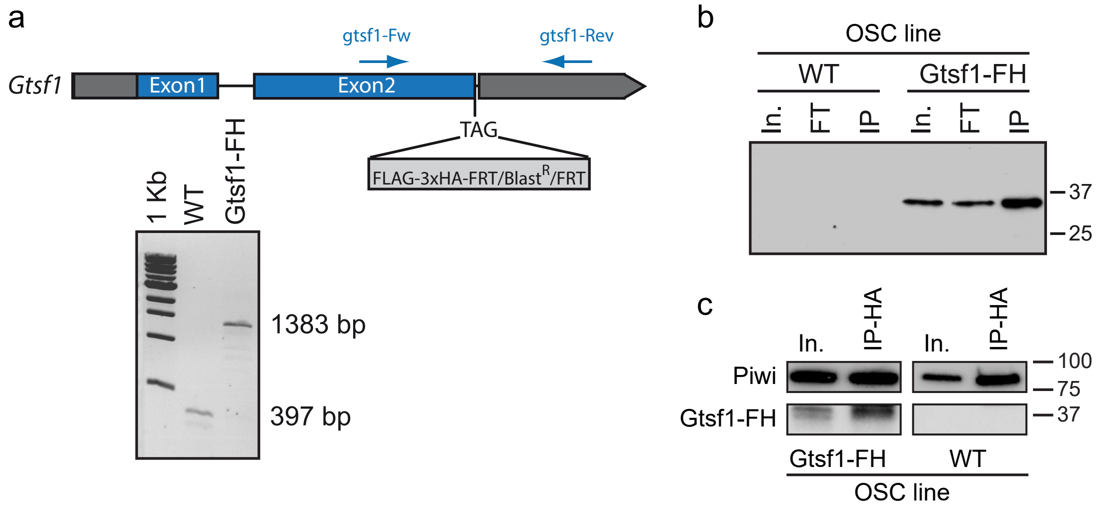

Supplementary Figure 2: CRISPR-Cas9 generation of endogenous FLAG-3xHA-tagged Gtsf1 in OSCs.

a) Schematic representation of the insertion of the FLAG-3xHA tag at the C-terminus of the *Gtsf1* gene and of the position of the primers used to amplify genomic DNA (top). Validation of genome editing is shown by ethidium bromide staining of the fragments obtained by PCR amplification of genomic DNA from wild type (WT) or genome-edited (Gtsf1-FH) OSCs (bottom). b) Immunoprecipitation (IP) with anti-HA antibody of nuclear extracts from wild type (WT) or genome-edited (Gtsf1-FH) OSCs and western blotting of input and output after IP. FT, flow through after IP. Western blotting was performed with anti-HA antibody. Molecular masses are indicated on the right. c) Non-specific binding of Piwi to anti-HA-conjugated agarose beads in wild-type OSCs. IP with anti-HA antibody of nuclear extracts from Gtsf1-FH or WT and western blotting of input and IP using anti-HA or Piwi antibody.

Source data: uncropped blot images are provided in Supplementary Data set 1.

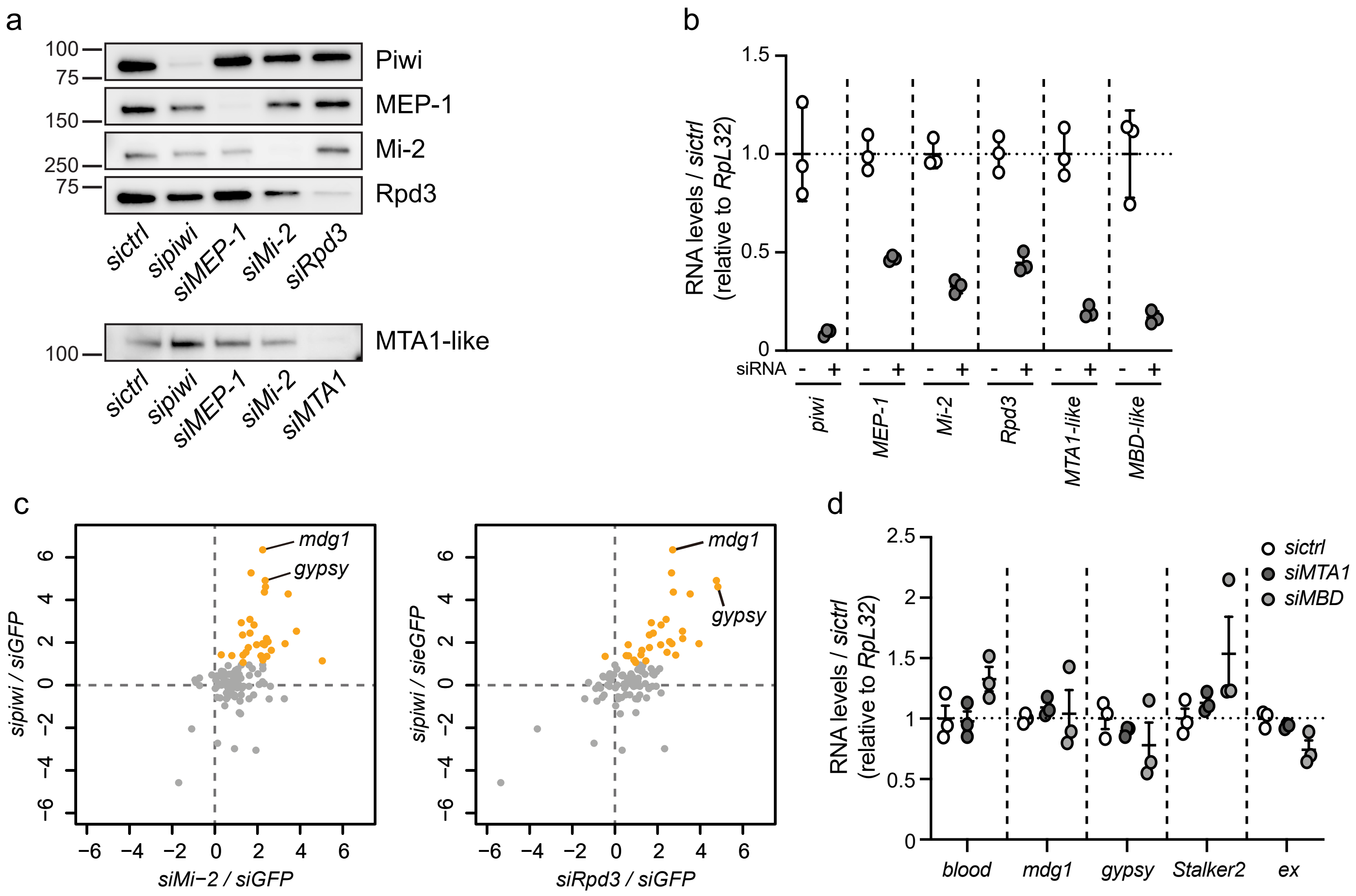

Supplementary Figure 3: The somatic depletion of Mi-2 and Rpd3 results in TE derepression *ex vivo* **independently of the core NuRD subunits MTA1-like and MBD-like.**

a-b) Validation of the RNAi efficiencies in OSCs. The western blots (a) show the protein levels of Piwi, MEP-1, Mi-2, Rpd3 and MTA1-like upon siRNA-mediated knockdown. b) Dots show RT-qPCR fold changes (relative to sictrl) in genes activity upon presence (+) or absence (-) of the corresponding siRNA for indicated genes. RNA levels were quantified relative to *Rpl32*. Data are presented as mean values +/- sd from n=3 biologically independent samples. c) Scatter plots comparing the fold changes (log2) in TE expression (mRNA-seq) caused by the indicated KDs. TEs for which the expression level differed from control (siGFP) by more than two fold in Piwi-KD (Piwi–piRNA-targeted TEs) were plotted in orange. d) RT-qPCR fold changes in steady-state RNA levels of four endogenous TEs and of the *expanded* (*ex*) gene upon MTA1-like (*siMTA-1*) and MBD-like (*siMBD*) knockdowns using siRNAs. RNA levels (mean ± s.d. from n=3 biologically independent samples) were quantified relative to *Rpl32* and normalized to control knockdown.

Source data for panel b, d: Supplementary Data set 7 and uncropped blot images are provided in Supplementary Data set 1.

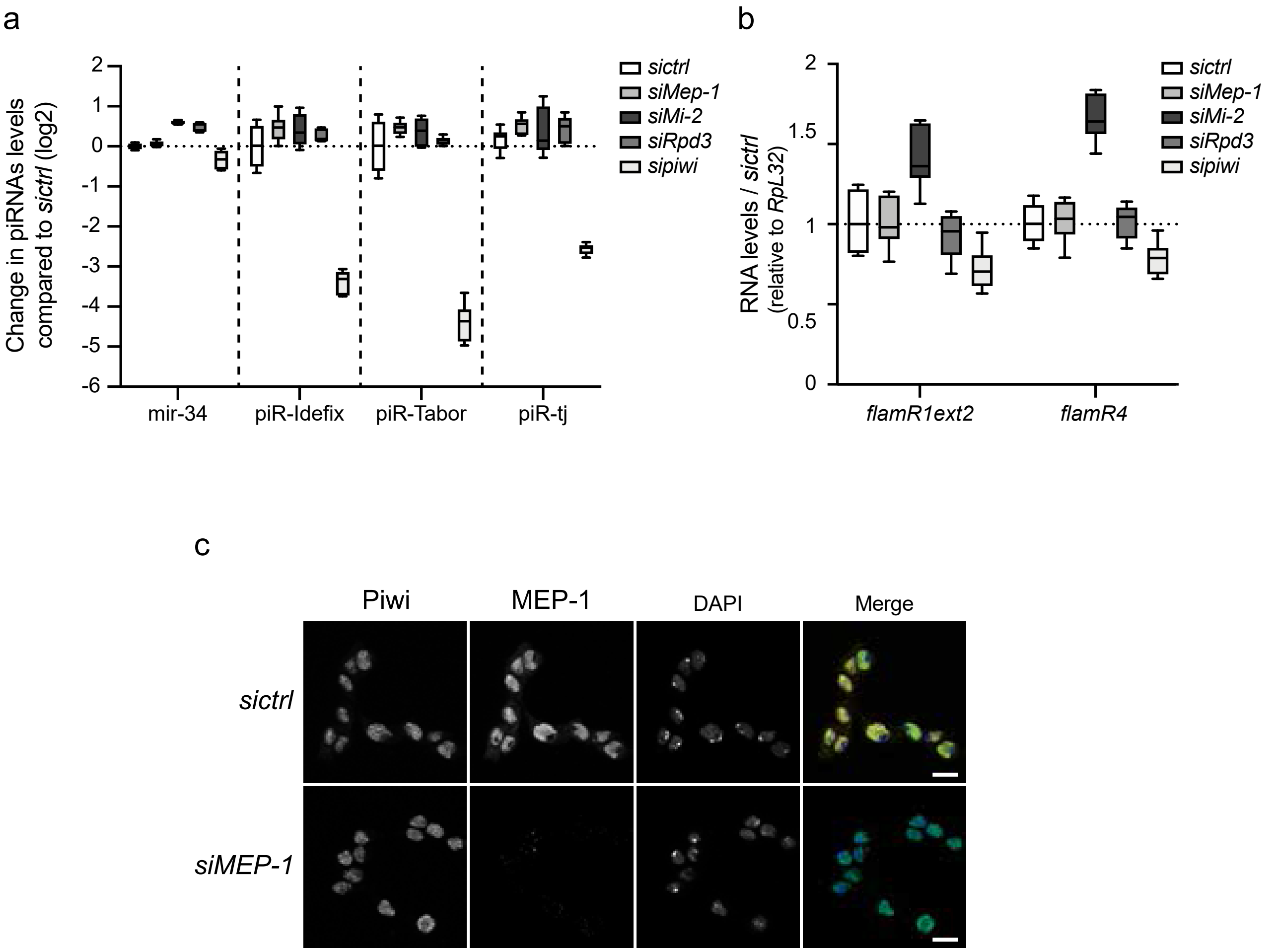

Supplementary Figure 4: The knockdown of MEP-1, Mi-2 and Rpd3 does not affect piRNA biogenesis in OSCs.

a) Box plots showing fold changes (relative to sictrl) in the levels of three major piRNAs (targeting *Idefix, Tabor* and *traffic jam* (*tj*)) in OSCs, upon transfection of indicated siRNAs. Box plots display median (line), first and third quartiles (box) and highest / lowest value within 1.5x interquatile range (whiskers) for n=6 values calculated over three biologically independent samples. b) Box plots showing RT-qPCR fold changes (relative to *sictrl*) in the level of transcripts produced by the *flamenco* piRNA cluster in OSCs upon silencing with the indicated siRNAs. Two different primer sets, flamR1ext2 and flamR4 were used. Box plots are defined as in a) c) Immunolabelling with anti-Piwi and anti-MEP-1 antibodies of OSCs transfected with the indicated siRNAs shows that efficient MEP-1 silencing does not affect the nuclear localization of Piwi. DAPI staining shows the nuclei. Scale bars, 10µm.

Source data for panel a, b: Supplementary data set 8.

Supplementary Methods

CRISPR/Cas9 DmGtsf1 genome editing

CRISPR/Cas9 genome editing of DmGtsf1 in OSC was adapted from[1]. The template plasmid pMH3 (#52528 Addgene) for HR-mediated protein tagging was kindly provided by K. Förstemann. The eGFP sequence from pMH3 was replaced by a FLAG-HA(x3) tag sequence using the *Xho*I/*Spe*I restriction sites. The FLAG-HA(x3) tag was amplified from the pAFHW-Piwi vector (kindly provided by J. Brennecke) using the following primers:
5'-tactcgagGATTACAAGGATGACGATGACA and
5'-taactagttcaAGCGTAATCTGGAACGTCA. The resulting plasmid was used as a template to generate a PCR product that contains *Gtsf1* homology arms (capital letters in the primer sequence) using the following primers:
5'-AGAAGCCGCGGAAGGCCAAGGCTCGCGCGGATCTTCGTCCCACTCCCTACGAGCACAGGAGGCCATACTCAAGGCGCCAGggatcttccggatggctcgag and 5'-CCGATAAAATGTTTTGGTTTTTGTTCTTTGGCAGTGGGAGTAATTCTTGATGAACACGAAgaagttcctattctctagaaagtataggaacttccatatg.
To obtain the *Gtsf1* small RNA guide-expressing pU6-BbsI-Gtsf1-sgRNA plasmid, two oligonucleotides (5'-CTTCGAACTACTGGCGCCTTGAGTA and 5'-AAACTACTCAAGGCGCCAGTAGTTC) that contain the *Gtsf1* sequence were phosphorylated, annealed and cloned in the pU6-BbsI-chiRNA vector (#45946 Addgene) using the *Bbs*I restriction site.
$3x10^6$ OSCs were electroporated with a mixture containing 1µg of the Cas9-encoding pRB14 vector (kindly provided by K. Förstemann, #52522 Addgene), 1 µg of pU6-BbsI-Gtsf1-sgRNA, 1 µg of the PCR product described above in 100µl of Mirus Ingenio® solution, using Amaxa Nucleofector II (program setting T-029). After transfection, OSCs were cultured in a medium supplemented with 50 µM SCR7 for 4 days, followed by selection with 25 µg/ml blasticidin for 1 month before testing the homologous recombination by PCR.

GST-pull down assays

pGEX5X-1-DmGtsf1 (kindly provided by M. Siomi's laboratory) and pGEX4T were transfected in the E. coli strain BL21 (DE3). Protein expression was induced by addition of 1 mM IPTG at 18°C overnight. Purified GST-DmGtsf1 and GST were immobilized on glutathione Sepharose 4 fast flow (GE Healthcare) and incubated with OSC nuclear extracts at

room temperature (RT) for 90min. To prepare nuclear extracts, OSCs were washed with 1X PBS and lysed in 10mM Tris-HCL pH 7.3, 1.5mM MgCl2, 10mM KCL, 1mM DTT, 0.2mM PMSF by 15 strokes with a Dounce homogenizer (pestle A). Lysates were centrifuged at 1,500xg at 4 °C for 15min. Nuclear pellets were washed with 1X PBS and resuspended in 20mM Tris-HCl (pH 8.0), 100mM KCl, 5mM MgCl2, 2mM DTT, 0.1% Triton X-100 and EDTA-free protease inhibitor cocktail (Thermo scientific) at 4 °C for 30min, followed by 13 strokes with a Dounce homogenizer (pestle B).

Bound proteins were eluted in a cold buffer containing 20 mM Tris-HCl (pH 8.0), 100 mM KCl, 5 mM MgCl2, 2 mM DTT, 0.1% Triton X-100, and EDTA-free protease inhibitor cocktail, resolved on NuPAGE Novex 4%–12% Bis-Tris gels, and stained with Coomassie blue. Bands were cut from the gel and proteins were identified by mass spectrometry (Taplin Biological Mass Spectrometry Facility, Harvard Medical School)

Methods for Protein Sequence Analysis by LC-MS/MS. (Taplin Biological Mass Spectrometry Facility)

Excised gel bands were cut into approximately 1 mm^3 pieces. Gel pieces were then subjected to a modified in-gel trypsin digestion procedure[2]. Gel pieces were washed and dehydrated with acetonitrile for 10 min. followed by removal of acetonitrile. Pieces were then completely dried in a speed-vac. Rehydration of the gel pieces was with 50 mM ammonium bicarbonate solution containing 12.5 ng/µl modified sequencing-grade trypsin (Promega, Madison, WI) at 4ºC. After 45 min., the excess trypsin solution was removed and replaced with 50 mM ammonium bicarbonate solution to just cover the gel pieces. Samples were then placed in a 37ºC room overnight. Peptides were later extracted by removing the ammonium bicarbonate solution, followed by one wash with a solution containing 50% acetonitrile and 1% formic acid. The extracts were then dried in a speed-vac (~1 hr). The samples were then stored at 4ºC until analysis. On the day of analysis, the samples were reconstituted in 5 - 10 µl of HPLC solvent A (2.5% acetonitrile, 0.1% formic acid). A nano-scale reverse-phase HPLC capillary column was created by packing 2.6 µm C18 spherical silica beads into a fused silica capillary (100 µm inner diameter x ~30 cm length) with a flame-drawn tip[3]. After equilibrating the column, each sample was loaded via a Famos auto sampler (LC Packings, San Francisco CA) onto the column. A gradient was formed and peptides were eluted with increasing concentrations of solvent B (97.5% acetonitrile, 0.1% formic acid). As peptides eluted they were subjected to electrospray ionization and then entered into an LTQ Orbitrap Velos Pro ion-trap mass

spectrometer (Thermo Fisher Scientific, Waltham, MA). Peptides were detected, isolated, and fragmented to produce a tandem mass spectrum of specific fragment ions for each peptide. Peptide sequences (and hence protein identity) were determined by matching protein databases with the acquired fragmentation pattern by the software program Sequest (Thermo Fisher Scientific, Waltham, MA)[4]. All databases include a reversed version of all the sequences and the data were filtered to between a one and two percent peptide false discovery rate.
The values presented in the Supplementary Data1 and the Supplementary Fig.1b are the sum raw intensity values to which a pseudo-count of 0.1 has been added. This allows to calculate a ratio [intensityGST-gtsf1/Mass] / [intensityGST/Mass] even for the proteins not detected in the GST control experiment.

Dual-luciferase co-immunoprecipitation in S2R+ cells

We adapted the protocol described in [39], with minor modifications. S2R+ cells from DGRC were cultivated at 25°C in Schneider medium complemented with 10% Foetal Bovine Serum (Eurobio) and 1% penicillin-streptomycin (Gibco). The cells were seeded at 4x105 cells/well in 24-well plates and transfected 24h after with two plasmids (0.15µg each), in quadruplicates using Effectene (301425, Qiagen). After 48h, cells were washed with PBS and lysed with 250 µl HNTG buffer (20 mM Hepes pH7.9, 150 mM NaCl, 1 mM MgCl2, 1 mM EDTA, 1% triton, 10% glycerol) supplemented with proteases inhibitors (Halt inhibitor cocktail, Thermo Scientific). Highbinding 96-well plates (655074 Lumitrac, Greiner) were used for IP. The plates were coated with 10 µg/ml M2 anti-FLAG antibodies (F1804 Sigma-Aldrich) overnight and blocked with BSA 3 %; Sucrose 5 %; Tween 20 0,5 %; PBS for 1h. IPs were performed with 100 µl of lysate/well overnight at 4°C. IPs were washed 5 times with 200 µl HNTG/well for 5 min at 20° on a thermomixer (Eppendorf) with intermittent shacking. In parallel, a plate "Input" was realised with 10 µl of lysate/well without antibody. Firefly luciferase (FFL) and Renilla luciferase (RL) activities were then quantified with DUAL luciferase reporter assay (Promega) using 50 µl of reagents/well and an InfiniteF200 reader (TECAN). The co-IP efficiency between FFL and RL fusion proteins was calculated by: (IP RL / Input RL) / (IP FFL / Input FFL). For each bait tested fused to FFL, a negative control co-IP using a RL-mCherry prey was performed and used as reference to calculate a normalized co-IP efficiency.

Quantitative ChIP

ChIP on ovaries: Fly ovaries were dissected into cold Schneider's insect medium (Sigma) and homogenized in 500 µL buffer A (60 mM KCl, 15 mM NaCl, 15 mM Hepes, 0.5% Triton-X100, 10 mM sodium butyrate, complete EDTA-free protease inhibitor tablets (Roche)) with 1.8% formaldehyde (Thermo Scientific) at room temperature (RT) using a Douncer with pestle ''tight.'' Total time for homogenization and cross-linking was exactly 10 min. Glycine was added to 125 mM final, and samples were incubated 3 min at RT and then chilled on ice. Samples were centrifuged (5 min, 4000xg at 4°C) and pellets were resuspended in buffer A with 125 mM glycine, incubated 5 min with agitation, and then centrifuged. The washing step was repeated two times with 1 ml buffer A and another time with 1 ml lysis buffer (140 mM NaCl, 15 mM Hepes, 1 mM EDTA, 0.5 mM EGTA, 1% Triton-X100, 0.1% sodium deoxycholate, 10 mM sodium butyrate, complete EDTA-free protease inhibitor). Pellets were resuspended in 200 µL lysis buffer with 0.5% SDS, 0.5% N-laurosylsarcosine and incubated 3 h at 4°C on a rotating wheel. Samples were sonicated with a Bioruptor (Diagenode; high level, 30 sec on, 30 sec off, repeated 12 times with resuspension after six times) and centrifuged (10 min, 20000xg at 4°C). Lysates were then diluted to 0.1% SDS with lysis buffer. Dynabeads protein G (Invitrogen) were washed in PBS-0.1 mg/mL BSA three times, added to lysates and incubated 3 h at 4°C on a rotating wheel. The following steps were similar to which described in ChIP of OSCs.

ChIP of OSCs: cells were seeded and transfected as above. OSCs from 2 wells of a 6-well plate were fixed in 1 ml M3 medium with 1.8% formaldehyde at RT for 10min. Glycine was added to 125 mM final concentration, and samples were incubated at RT for 5 min, and then chilled on ice. OSCs were rinsed in cold 1X PBS supplemented with 0.1 mg/ml pefabloc SC (Roche). Samples were centrifuged (500xg at 4°C for 7 min), and pellets were washed in 1 ml buffer I (10 mM Hepes pH 7.6, 0.25% Triton-X100, 10 mM EDTA pH 8, 0.5 mM EGTA pH 7.5, EDTA-free protease inhibitor), followed by one wash in buffer II (200 mM NaCl, 10 mM Hepes pH 7.6, 1 mM EDTA pH 8, 0.5 mM EGTA, EDTA-free protease inhibitor). Pellets were then resuspended in 300 µl lysis buffer (140 mM NaCl, 15 mM Hepes pH 7.6, 1 mM EDTA pH 8, 0.5 mM EGTA, 1% Triton-X100, 0.1% SDS, 0.5 mM DTT, 0.5% N-laurosylsarcosine, 0.1% sodium deoxycholate, 10 mM sodium butyrate, EDTA-free protease inhibitor) and incubated at 4°C on a rotating wheel for 30min. Samples were sonicated with a Bioruptor (Diagenode, high level, 30sec on, 30sec off, repeated 20 times with resuspension after 10 times) and centrifuged (15,000xg at 4°C for 5min). Supernatants were collected and incubated at 4°C with Dynabeads protein G (Invitrogen) for 1h. Supernatants were then incubated at 4°C on a rotating wheel with

2 µl of anti-H3K9me3 (ab8898; Abcam), 2 µl of anti-histone H3 (ab1791; Abcam) or 2 µl of anti-H3K9ace antibodies (ab4441; Abcam) overnight. After incubation, 30 µl Dynabeads protein G was added at 4°C for 1h. Beads were then washed twice in RIPA buffer (150 mM NaCl, 50 mM Tris pH 8.1, 0.1% SDS, 1% NP40), once in high-salt buffer (500 mM NaCl, 50 mM Tris pH 8.1, 0.1% SDS, 1% NP40), once in LiCl buffer (250 mM LiCl, 50 mM Tris pH 8.1, 0.5% sodium deoxycholate, 1% NP40), and three times in TE (10 mM Tris pH 8, 0.1 mM EDTA). Chromatin was eluted by adding 100 µl of elution buffer I (10 mM EDTA, 1% SDS, 50 mM Tris pH 8) and incubated at 65°C under vigorous shaking for 15 min. Elution was repeated with 150 µl of elution buffer 2 (0.67% SDS in TE). The two supernatants were pooled and supplemented with SDS and NaCl to obtain a final concentration of 1% SDS and 200 mM NaCl. Cross-linking of each immunoprecipitated complex was reversed by incubating the eluates at 65°C overnight. After digestion with RNase A (50 µg/ml, at 37°C for 1h) and with proteinase K (40 µg/ml, at 55°C for 2h), DNA was purified by phenol-chloroform extraction and precipitated with ethanol. Precipitated DNA was used as template for quantitative PCR with specific primers (Supplementary Table 1). Three biological replicates were used for ChIP experiments, and two technical replicates for each OSC or ovaries sample were quantified by qPCR.

Drosophila husbandry and strains

Ovaries were dissected from 5 day-old females reared on standard cornmeal/yeast diet. Drosophila stocks are described in Supplementary Table 3. Drosophila genotypes and breeding temperatures are specified as follows:

Fig. 4a-c: Females were continuously reared at 18°C except for the 5-day treatment at 25°C which was performed just before dissection in the GAL80ts experiments (Fig. 4a, right panels, and Fig. 4c). They were all non-Cy daughters of RNAi males crossed with females originating from either the tj-GAL4, gypsy-lacZ/CyO; ZAM-lacZ, tubP>GAL80ts strain (GAL80 experiments) or the tj-GAL4, gypsy-lacZ/CyO strain.

Fig. 6c-e: Females were continuously reared at 25°C. All flies shared the same genotype for chromosome 3: P{GAL4::VP16-nos.UTR}/ LacO-nos>GFP-Piwi. The genotype for chromosome 2 was : LacI : pUASp>LacI/+ , LacI-MEP-1: pUASp>LacI-MEP-1/+.

LacI-MEP-1 transgenic flies

The construct expressing the DNA-tethering LacI-MEP-1 fusion protein was obtained by replacing the *silencio* sequence in the UASP-LacI-silencio plasmid (gift from J. Brennecke)

with MEP-1 cDNA (BDGP_cDNA: RE60032) after correction of four mismatches with the reference sequence. The resulting PattB-UASP-LacI-MEP-1 plasmid was used by BestGene to generate the corresponding transgenic fly stock with PhiC31-mediated transformation into attP40.

Supplementary Tables

Supplementary Table 1: Primer list

Genes	Application	Forward primer	Reverse primer	Ref
RpL32	qPCR	CAGCTTCAAGATGACCATC	GTTCGATCCGTAACCGATGT	
light	qPCR	GCTAGGCAATGACAAAGTCCTTTGGG	GCATTCGTCTGAAGTCGGCAGATAG	
1360-element	qPCR	GGAGCTCTGCGTATAGCCAACTT	ACCTAAACCGCCGAGTCCTG	
MEP-1	qPCR	CTACATCAAGGATCTGGAGCAG	AATCCCTGATCCGTAAAGAACC	
Mi-2	qPCR	ATGAAGAGCGATGTGTCCC	ATTGTGCCATCAATTGAGCTG	
piwi	qPCR	GCGCTTTTAACCACCGTTTA	GGTCTCTGAAGTGCCTTTGC	
Gtsf1	PCR (Supplementary fig. 2a)	TCAGCGAAGACACCAGACAC	TAACAATTTGGCCGTGGAAT	
LacZ	qPCR	TGGCAGATGCACGGTTACGA	TCAGACGGCAAACGACTGTCC	
expanded	qPCR	CGTGTGCAATTGTTCTGGTG	CTTTCTGACCTCTTCCACTTCC	
krimper	qPCR	TACGATCCCAAGCTGAATGG	GCCGATTTCAAAATGCACTG	
	ChIP	GGCGTTGAAAGCTTCGATTA	ATTATTGAACAGCCGCCAAA	47
MBD-like	qPCR	TGCCTCGATCTTCAAGCAACC	TAGTCGTTCCAGTCGCTTCTCC	
MTA1-like	qPCR	CATCCTCCATTTCCATACACC	CTGATCCTAAGTCGAATGCC	
LacO-GFP reporter	qPCR	CGCGAATTCGGATCTAGTG	GGAATAATGTAGTTTTCCAGCGATA	
GFP	qPCR	GACGTAAACGGCCACAAGTTC	TTGCCGGTGGTGCAGAT	

Transposons	Application	Forward primer	Reverse primer
Tabor	qPCR	ACGTTGTTCACGACATTAGCCG	GGGTTGGTTCGGATCTGACG
gypsy	qPCR	CTTCACGTTCTGCGAGCGGTCT	CGCTCGAAGGTTACCAGGTAGGTTC
	ChIP	GGCTCATTGCCGTTAAACAT	TCTTTCGCTGAGGTTCGTCT
mdg1	qPCR	GCCCAAAAATTTCAGGAACA	TTACGAGTGCACTTGCGATT
	ChIP	ATGCGAATTCAGGATGTACG	AGGGAGATCTCTTGTGACAGC
blood	qPCR	GGGAATTCTAAACAGCGACAAC	CCCATTACCACACTCTACTCAC
Stalker2	qPCR	CCCAAGAGGTGGAGAAACTG	ATGTGCCTGCTTGCATGATA

piRNA	Application	Forward primer	Reverse primer	Ref
Idefix	qPCR	GTTCCCAAACGATTGCCA	TCCAGTTTTTTTTTTTTTTTTAAACTAC	
Tabor	qPCR	GCAGTAAACCTATTGAATTTATTG	CCAGTTTTTTTTTTTTTTTTAAGGGT	
traffic jam	qPCR	GTTCAAGAGAAGTGCATTCC	GTCCAGTTTTTTTTTTTTTTTTGGTAA	51

miRNA	Application	Forward primer	Reverse primer	Ref
miR-14	qPCR	CAGTCAGTCTTTTTCTCTCT	GTCCAGTTTTTTTTTTTTTTTTATAGG	51
miR-34	qPCR	TGGCAGTGTGGTTAGCT	TCCAGTTTTTTTTTTTTTTTTCACAA	51

flamenco region	Application	Forward primer	Reverse primer	Ref
flamR1ext2	qPCR	TCAAGGAAAACGTGGGAAAG	AGGAGGAAACCCTCCAGAAA	[5]
flamR4	qPCR	CAGATTACCATTTGGCTATGAGGATCAGAC	TGGTGAAATACCAAAGTCTTGGGTCAAC	[51]

Targeted genes	siRNA	Sequence (5’->3’)	Ref
-	sictrl	GAACUAUUCACGACAUAUG	
MEP-1	siMEP-1	UAAUCUGUGAGUCUUCGUC	
Mi-2	siMi-2	AUAUCGUUGUGGGGAUUCCA	
piwi	sipiwi	CACCUUCACGCCUGGGAGC	[46]
Rpd3	siRpd3	GCAGAUGCAGCGUUUCAAU	
MBD	siMBD	GGAGGACGACAUACGCAAA	
MTA1-like	siMTA-1	CGACAAGGAGCUCACAAUA	

Genes	Application	Forward primer	Reverse primer
piwi	PCR for cloning Dual luciferase	AAAAAAGCAGGCTCCGCGGCCGCCCCCTCACCATGGCTGATGATCAGGGACGTG	AAAGCTGGGTCGGCGCGCCCACCCTTTAGATAATAAAACTTCTTTTCGAGC
Gtsf1	PCR for cloning Dual luciferase	AAAAAAGCAGGCTCCGCGGCCGCCCCCTTCACCATGGTTTATTGCCCGTACAACA	AAAGCTGGGTCGGCGCGCCCACCCTTCTGGCGCCTTGAGTATGGC
MEP-1	PCR for cloning Dual luciferase	AAAAAAGCAGGCTCCGCGGCCGCCCCCTTCACCATGGGAACTGAAGTTGATGTCGTTTTG	AAAGCTGGGTCGGCGCGCCCACCCTTATCTATGACATGACTCTCCAT
MEP-1ΔCt	PCR for cloning Dual luciferase	AAAAAAGCAGGCTCCGCGGCCGCCCCCTTCACCATGGGAACTGAAGTTGATGTCGTTTTG	AAAGCTGGGTCGGCGCGCCCACCCTTGGCGGTGGCAGTGCCCACAA
MEP-1ΔNt	PCR for cloning Dual luciferase	AAAAAAGCAGGCTCCGCGGCCGCCCCCTTCACCATGGGAAAGCGATGCGAGTTCTGCAAC	AAAGCTGGGTCGGCGCGCCCACCCTTATCTATGACATGACTCTCCAT
Su(var)2-10	PCR for cloning Dual luciferase	AAAAAAGCAGGCTCCGCGGCCGCCCCCTTCACCATGGGAGTGCAGATGCTTCGAGTGGTCG	AAAGCTGGGTCGGCGCGCCCACCCTTTTGCAAAAAGGGATCCAACGTACCG
SetDB1	PCR for cloning Dual luciferase	AAAAAAGCAGGCTCCGCGGCCGCCCCCTTCACCATGGGATCTGGGCAGCCAACAGC	AAAGCTGGGTCGGCGCGCCCACCCTTGAGCAGACGAAGGCGGC
Cherry	PCR for cloning Dual luciferase	AAAAAAGCAGGCTCCGCGGCCGCCCCCTTCACCATGGTGAGCAAGGGCGAGGAG	AAAGCTGGGTCGGCGCGCCCACCCTTCTTGTACAGCTCGTCCATGCCG

Supplementary Table 2: Statistics and Reproducibility

Figure	Comparison	Test	F-values	t-values	Degrees of freedom	p-value
5b		ANOVA	41.62		4	1.05e-10
5b	sictrl/siMEP-1	Two-tailed Student’s t-test		9.7682	10	1.97e-06
5b	sictrl/siMi-2	Two-tailed Student’s t-test		5.9133	10	0.0001484
5b	sictrl/sipiwi	Two-tailed Student’s t-test		7.298	10	2.607e-05
5b	sictrl/siRpd3	Two-tailed Student’s t-test		12.689	10	1.725e-07

5d		ANOVA	14.769		2	0.004811
5d	sictrl/siMEP-1	Two-tailed Student's t-test		3.7571	4	0.01983
5d	sictrl/sipiwi	Two-tailed Student's t-test		4.6784	4	0.009459
6e	LacI H3K9me3/ LacI-MEP-1 H3K9me3	Two-tailed Student's t-test		22.6	5	0.000003
6e	LacI H3K9ace/ LacI-MEP-1 H3K9ace	Two-tailed Student's t-test		3.43	4	0.026472

Figure	1a	1d	2a	2b	6a	6c	Sup. 1b	Sup. 2a	Sup. 2b	Sup. 2c	Sup. 3a	Sup. 4c
Reproducibility	2x	5x	2x	2x	2x	3x	1x	1x	2x	5x	2x	2x

Supplementary Table 3: Drosophila stocks

Drosophila Stocks		
D. melanogaster: RNAi of Piwi: y[1] sc[*] v[1]; P{y[+t7.7] v[+t1.8]=TRiP.HMS00606}attP2	Bloomington Drosophila Stock Center	BDSC#33724
D. melanogaster: RNAi of Mi-2: y[1] v[1]; P{y[+t7.7] v[+t1.8]=TRiP.HMC03329}attP40	Bloomington Drosophila Stock Center	BDSC#51774
D. melanogaster: RNAi of MEP-1: y[1] sc[*] v[1]; P{y[+t7.7] v[+t1.8]=TRiP.HMC05187}attP40	Bloomington Drosophila Stock Center	BDSC#62180
D. melanogaster: RNAi of Rpd3: y[1] sc[*] v[1] sev[21]; P{y[+t7.7] v[+t1.8]=TRiP.GL01005}attP40	Bloomington Drosophila Stock Center	BDSC#36800
D. melanogaster: RNAi of White: y[1] v[1]; ; P{TRiP.HMS00017}attP2	Bloomington Drosophila Stock Center	BDSC#33623
D. melanogaster: ovarian somatic driver and gypsy piRNA sensor: hs-hid(Y); tj-GAL4, gypsy-lacZ/CyO;;	VDRC	VDRC#313222
D. melanogaster: ovarian germline driver: w[1118] ; ; P{GAL4::VP16-nos.UTR}CG6325^{MVD1}	Our lab collection	N/A
D. melanogaster: conditional somatic driver and gypsy piRNA sensor: ; tj-GAL4, gypsy-lacZ/CyO; ZAM-lacZ, tubP>GAL80^{ts}	Our lab collection	N/A
D. melanogaster: expression of LacI-MEP-1 fusion protein: y w; pUASp>LacI-MEP-1 [attP40]	This paper	N/A
D. melanogaster: expression of LacI control protein: ; pUASp>LacI/CyO ; Ki/Sb Ser	W. Theurkauf	N/A
D. melanogaster: source of LacO-GFP reporter transgene: ; pUASp>LacI-Panx [attP40]/CyO; lacO-nos>GFP-Piwi (with intron) [attP2]/TM3, Ser	VDRC	VDRC#313400

Supplementary References

1. Böttcher, R. *et al.* Efficient chromosomal gene modification with CRISPR/cas9 and PCR-based homologous recombination donors in cultured Drosophila cells. *Nucleic Acids Res.* **42**, e89 (2014).

2. Shevchenko, A., Wilm, M., Vorm, O. & Mann, M. Mass spectrometric sequencing of proteins silver-stained polyacrylamide gels. *Anal. Chem.* **68**, 850–858 (1996).
3. Peng, J. & Gygi, S. P. Proteomics: the move to mixtures. *J. Mass Spectrom. JMS* **36**, 1083–1091 (2001).
4. Eng, J. K., McCormack, A. L. & Yates, J. R. An approach to correlate tandem mass spectral data of peptides with amino acid sequences in a protein database. *J. Am. Soc. Mass Spectrom.* **5**, 976–989 (1994).
5. Haase, A. D. *et al.* Probing the initiation and effector phases of the somatic piRNA pathway in Drosophila. *Genes Dev* **24**, 2499–504 (2010).
