# Supplementary material for: The Mi-2 nucleosome remodeler and the Rpd3 histone deacetylase are involved in piRNA-guided heterochromatin formation
Source: Nat Commun. 2020 Jun 4;11:2818. doi: 10.1038/s41467-020-16635-5 (PMC7272611; doi:10.1038/s41467-020-16635-5)
Supplement: Supplementary file 3 — Reporting Summary [file 41467_2020_16635_MOESM3_ESM.pdf]

nature research

Corresponding author(s): Chambeyron Severine

Reporting Summary

Nature Research wishes to improve the reproducibility of the work that we publish. This form provides structure for consistency and transparency in reporting. For further information on Nature Research policies, see Authors & Referees and the Editorial Policy Checklist.

Statistical parameters

When statistical analyses are reported, confirm that the following items are present in the relevant location (e.g. figure legend, table legend, main text, or Methods section).

n/a	Confirmed	
☐	☒	The exact sample size (*n*) for each experimental group/condition, given as a discrete number and unit of measurement
☐	☒	An indication of whether measurements were taken from distinct samples or whether the same sample was measured repeatedly
☐	☒	The statistical test(s) used AND whether they are one- or two-sided<br>*Only common tests should be described solely by name; describe more complex techniques in the Methods section.*
☐	☒	A description of all covariates tested
☐	☒	A description of any assumptions or corrections, such as tests of normality and adjustment for multiple comparisons
☐	☒	A full description of the statistics including central tendency (e.g. means) or other basic estimates (e.g. regression coefficient) AND variation (e.g. standard deviation) or associated estimates of uncertainty (e.g. confidence intervals)
☐	☒	For null hypothesis testing, the test statistic (e.g. *F*, *t*, *r*) with confidence intervals, effect sizes, degrees of freedom and *P* value noted<br>*Give P values as exact values whenever suitable.*
☒	☐	For Bayesian analysis, information on the choice of priors and Markov chain Monte Carlo settings
☒	☐	For hierarchical and complex designs, identification of the appropriate level for tests and full reporting of outcomes
☒	☐	Estimates of effect sizes (e.g. Cohen's *d*, Pearson's *r*), indicating how they were calculated
☐	☒	Clearly defined error bars<br>*State explicitly what error bars represent (e.g. SD, SE, CI)*

Our web collection on statistics for biologists may be useful.

Software and code

Policy information about availability of computer code

Data collection	RNA-seq:R-packages implemented in R 3.2.1; bowtie2 (ver. 2.2.4) using default parameters<br>Mass Spectrometry: software program Sequest (Thermo Fisher Scientific, Waltham, MA)
Data analysis	Small guide design : http://tools.flycrispr.molbio.wisc.edu/targetFinder (Gratz et al. 2014)<br>Quantitative PCR: LightCycler480 Software- Roche- Release 1.5.1<br>Mass Spectrometry : https://taplin.med.harvard.edu/home<br>RNA-seq analyses: piPipes pipeline (https://github.com/bowhan/piPipes) and BEDTools genomecov

For manuscripts utilizing custom algorithms or software that are central to the research but not yet described in published literature, software must be made available to editors/reviewers upon request. We strongly encourage code deposition in a community repository (e.g. GitHub). See the Nature Research guidelines for submitting code & software for further information.

Data

Policy information about availability of data

All manuscripts must include a data availability statement. This statement should provide the following information, where applicable:

- Accession codes, unique identifiers, or web links for publicly available datasets
- A list of figures that have associated raw data
- A description of any restrictions on data availability

Figure 3a, b and Supplementary figure 3c : Data are accessible in the NCBI Gene Expression Omnibus (GEO; https://www.ncbi.nlm.nih.gov/geo/) under the accession number GSE112972.
Supplementary Fig. 1b: The mass spectrometry data have been analyses by Taplin MS facility and the RAW data have been deposited to the ProteomeXchange Consortium via PRIDE with the data set identifier PXD018749 and 10.6019/PXD018749.
All fly lines used in this study are listed in Supplementary Table 3 and come from Bloomington Drosophila Stock Center, VDRC, our collectionand W. Theurkauf lab.
Source data for all gel images are provided in Supplementary Data Set 1.

Field-specific reporting

Please select the best fit for your research. If you are not sure, read the appropriate sections before making your selection.

- [x] Life sciences
- [ ] Behavioural & social sciences
- [ ] Ecological, evolutionary & environmental sciences

For a reference copy of the document with all sections, see nature.com/authors/policies/ReportingSummary-flat.pdf

Life sciences study design

All studies must disclose on these points even when the disclosure is negative.

Sample size

No sample size calculation was performed. All experiments presented in this work were done on established experimental procedure in biology and in Drosophila genetics. Sample sizes are indicated throughout the manuscript and some details are listed below:

Figure 1a: GFP-Trap experiments were performed using 500 µg total proteins of OSC nuclear extracts. For Western blot analysis the equivalent of 250 µg proteins was loaded in the IPs in comparison to 2.5 µg proteins in the inputs (1%).
Figure 1c and 6b: LUMIER IPs were performed in 96-well microplates seeded with 400,000 S2 cells 24h prior to transfection and harvested 48h post-transfection in n=4 biological replicates.
Figure 1d and 2b: Piwi and MEP-1 endogenous IPs were performed using 500 µg total proteins of OSC nuclear extracts. For Western blot analysis the equivalent of 150 µg proteins was loaded in the IPs in comparison to 3 µg proteins in the inputs (2%).
Figure 2a: A total of 1 mg protein from OSC nuclear extract was applied to the Superose 6 HR 10/30 column. Fractions were precipitated with TCA, and the Western blots presented were obtained by loading one third of total proteins from each fraction.
Figure 3a,b: Total RNAs from 3000000 cells were isolated using ISOGEN◦ (Nippon Gene) then purification of poly-A RNAs. The sequencing was done using HiSeq2500 (Illumina).
Figure 3c, 4b-c, 5b-c, suppl Fig 3b, suppl Fig 3d, suppl Fig 4b: RT from ovaries or OSCs were performed on 500 ng total RNA in biological triplicates.
Figure 3d-f and 5d: Quantitative ChIP experiments were performed from 2 wells of confluent 6-well plates in biological triplicates.
Figure 6a: GFP-Trap were performed using 500 µg total proteins of OSC nuclear extracts, and the equivalent of 250 µg proteins was loaded in the IPs in comparison to 2.5 µg proteins in the inputs (1%). MEP-1 IPs were performed using 200 µg total proteins of OSC nuclear extracts and the totality of bound proteins was loaded for Western blot analysis in comparison to 4 µg proteins in the inputs (2%).

Data exclusions

No data were excluded for analyses.

Replication

All the experiments were performed using at least 3 independant samples (n=3) and the measurement for each sample was done twice (technical duplicate). The experiments have been done at least 2 times only the obtention of the stable cell line was done 1 time and the Mass spectrometry presented in Supplementary Fig. 1.

Randomization

Does not apply

Blinding

Blinding was not relevant to this study

Reporting for specific materials, systems and methods

Materials & experimental systems

n/a	Involved in the study
☐	☒ Unique biological materials
☐	☒ Antibodies
☐	☒ Eukaryotic cell lines
☒	☐ Palaeontology
☐	☒ Animals and other organisms
☒	☐ Human research participants

Methods

n/a	Involved in the study
☒	☐ ChIP-seq
☒	☐ Flow cytometry
☒	☐ MRI-based neuroimaging

Unique biological materials

Policy information about availability of materials

Obtaining unique materials: All Unique biological materials are available from the authors.

Antibodies

Antibodies used:

All antibodies used in this study are listed either in Methods section or in Suppelementary Information. Dilutions for Western blot are indicated here.
Rabbit polyclonal anti-Piwi (d-190) Santa Cruz Biotechnology Cat#sc98264 ; RRID:AB_1566910 (used at 1:3000 in WB)
Mouse monoclonal anti-Piwi G-1 Santa Cruz Biotechnology Cat#sc390946 (used at 1:3000 in WB)
Rabbit polyclonal anti-MEP-1 (gift from A. Brehm) (used at 1:2500 in WB)
Rabbit polyclonal anti-Mi-2 (gift from A. Brehm) (used at 1:2500 in WB)
Rabbit polyclonal anti-Rpd3 (gift from J. Kadonaga) (used at 1:5000 in WB)
Rabbit polyclonal anti-CAF1-p55 (gift from J. Kadonaga) (used at 1:10000 in WB)
Guinea pig polyclonal anti-MTA1-like (gift from C.P. Verrijzer) (used at 1:2000 in WB)
Rabbit polyclonal anti-Pc (gift from G. Cavalli) (used at 1:1500 in WB)
Mouse monoclonal anti-Egg (gift from M.C. Siomi) (used 1:5 in WB)
Mouse monoclonal anti-GFP Sigma-Aldrich Cat# 11814460001 ; RRID:AB_390913 (used at 1:2000 in WB)
Rat monoclonal anti-HA (3F10) Roche Cat#11867423001 ; RRID:AB_390918 (used at 1:1000 in WB)
Mouse monoclonal anti-FLAG (M2) Sigma-Aldrich Cat#F1804 ; RRID:AB_262044 (used at 1:1000 in WB)
Rabbit polyclonal anti-Histone H3 (tri methyl K9) Abcam Cat#ab8898 ; RRID:AB_306848
Rabbit polyclonal anti-Histone H3 (acetyl K9) Abcam Cat#ab4441 ; RRID:AB_2118292
Rabbit polyclonal anti-Histone H3 Abcam Cat#ab1791 ; RRID:AB_302613
Alexa Fluor® 555 donkey anti-rabbit IgG Molecular Probe Cat#A31572 ; RRID:AB_162543
Alexa Fluor® 488 donkey anti-mouse IgG Molecular Probe Cat#A21202 ; RRID:AB_141607
Alexa Fluor® 647 goat anti-mouse IgG Thermo Fisher Scientific Cat#A21236 ; RRID:AB_2535805
EasyBlot anti Rabbit IgG (HRP) GeneTex Cat# GTX221666-01S, RRID:AB_10618575 (used at 1:2000 in WB)
EasyBlot anti Mouse IgG (HRP) GeneTex Cat# GTX221667-01, RRID:AB_10728926 (used at 1:2000 in WB)
Goat anti-Mouse IgG (H+L) Secondary Antibody, HRP Thermo Fisher Scientific Cat# A16066, RRID:AB_2534739 (used at 1:5000 in WB)
Goat anti-Rat IgG (H+L) Cross-Adsorbed Secondary Antibody, HRP Thermo Fisher Scientific Cat# A18871, RRID:AB_2535648 (used at 1:5000 in WB)
Anti-rabbit IgG, HRP-linked Antibody Cell Signaling Technology Cat# 7074, RRID:AB_2099233 (used at 1:5000 in WB)

Validation: Specifity of the antibodies are shown in Suppelementary Fig. 3 using RNAi experiments and Western blots or RT-qPCR.

Eukaryotic cell lines

Policy information about cell lines

Cell line source(s):
OSC cell line from Mikiko Siomi lab (https://www.s.u-tokyo.ac.jp/en/people/siomi_mikiko/)(Saito et al., 2009)
Schneider's S2-R+ cell line from the Drosophila RNAi Screening Center (DRSC), Harvard Medical School, Boston

Authentication: Does not apply

Mycoplasma contamination: Free of mycoplasma contamination.

Commonly misidentified lines (See ICLAC register): Does not apply

Animals and other organisms

Policy information about studies involving animals; ARRIVE guidelines recommended for reporting animal research

Laboratory animals	D. melanogaster: RNAi of Piwi: y[1] sc[*] v[1]; P{y[+t7.7] v[+t1.8]=TRiP.HMS00606}attP2 Bloomington Drosophila Stock Center<br>D. melanogaster: RNAi of Mi-2: y[1] v[1]; P{y[+t7.7] v[+t1.8]=TRiP.HMC03329}attP40 Bloomington Drosophila Stock Center<br>D. melanogaster: RNAi of MEP-1: y[1] sc[*] v[1]; P{y[+t7.7] v[+t1.8]=TRiP.HMC05187}attP40 Bloomington Drosophila Stock Center<br>D. melanogaster: RNAi of Rpd3: y[1] sc[*] v[1] sev[21]; P{y[+t7.7] v[+t1.8]=TRiP.GL01005}attP40 Bloomington Drosophila Stock Center<br>D. melanogaster: RNAi of White: y[1] v[1]; ; P{TRiP.HMS00017}attP2 Bloomington Drosophila Stock Center<br>D. melanogaster: ovarian somatic driver and gypsy piRNA sensor: hs-hid(Y); tj Gal4, gyspsy-lacZ/CyO;; VDRC<br>D. melanogaster: conditional somatic driver and gypsy piRNA sensor: ; tj-GAL4, gypsy-lacZ/CyO; ZAM-lacZ, tubP>Gal80ts Our lab collection<br>D. melanogaster: expression of LacI-MEP-1 fusion protein: y w; pUASp>LacI-MEP-1 [attP40] This paper<br>D. melanogaster: expression of LacI control protein: ; pUASp>LacI/CyO ; Ki/Sb Ser W. Theurkauf<br>D. melanogaster: source of LacO-GFP reporter transgene: ; pUASp>LacI-Panx [attP40]/CyO; lacO-nos>GFP-Piwi (with intron) [attP2]/TM3, Ser VDRC
Wild animals	The study did not involve wild animals.
Field-collected samples	The study did not involve field collected samples
