# Supplementary material for: The Mi-2 nucleosome remodeler and the Rpd3 histone deacetylase are involved in piRNA-guided heterochromatin formation
Source: Nat Commun. 2020 Jun 4;11:2818. doi: 10.1038/s41467-020-16635-5 (PMC7272611; doi:10.1038/s41467-020-16635-5)
Supplement: Supplementary file 4 — Description of Additional Supplementary Files [file 41467_2020_16635_MOESM4_ESM.pdf]

Description of Additional Supplementary Files

File Name: Supplementary Data 1
Description: The values are the sum raw intensity values to which a pseudo-count of 0.1 has been added. This allows to calculate a ratio [intensityGST-gtsf1/Mass] / [intensityGST/Mass] even for the proteins not detected in the GST control experiment.
