# Supplementary material for: The Mi-2 nucleosome remodeler and the Rpd3 histone deacetylase are involved in piRNA-guided heterochromatin formation
Source: Nat Commun. 2020 Jun 4;11:2818. doi: 10.1038/s41467-020-16635-5 (PMC7272611; doi:10.1038/s41467-020-16635-5)

Supplementary Data set 1 - Uncropped western blot and gel images.

«kD» indicates protein molecular weight marker and the square box indicates the cropped regions.

Fig1a

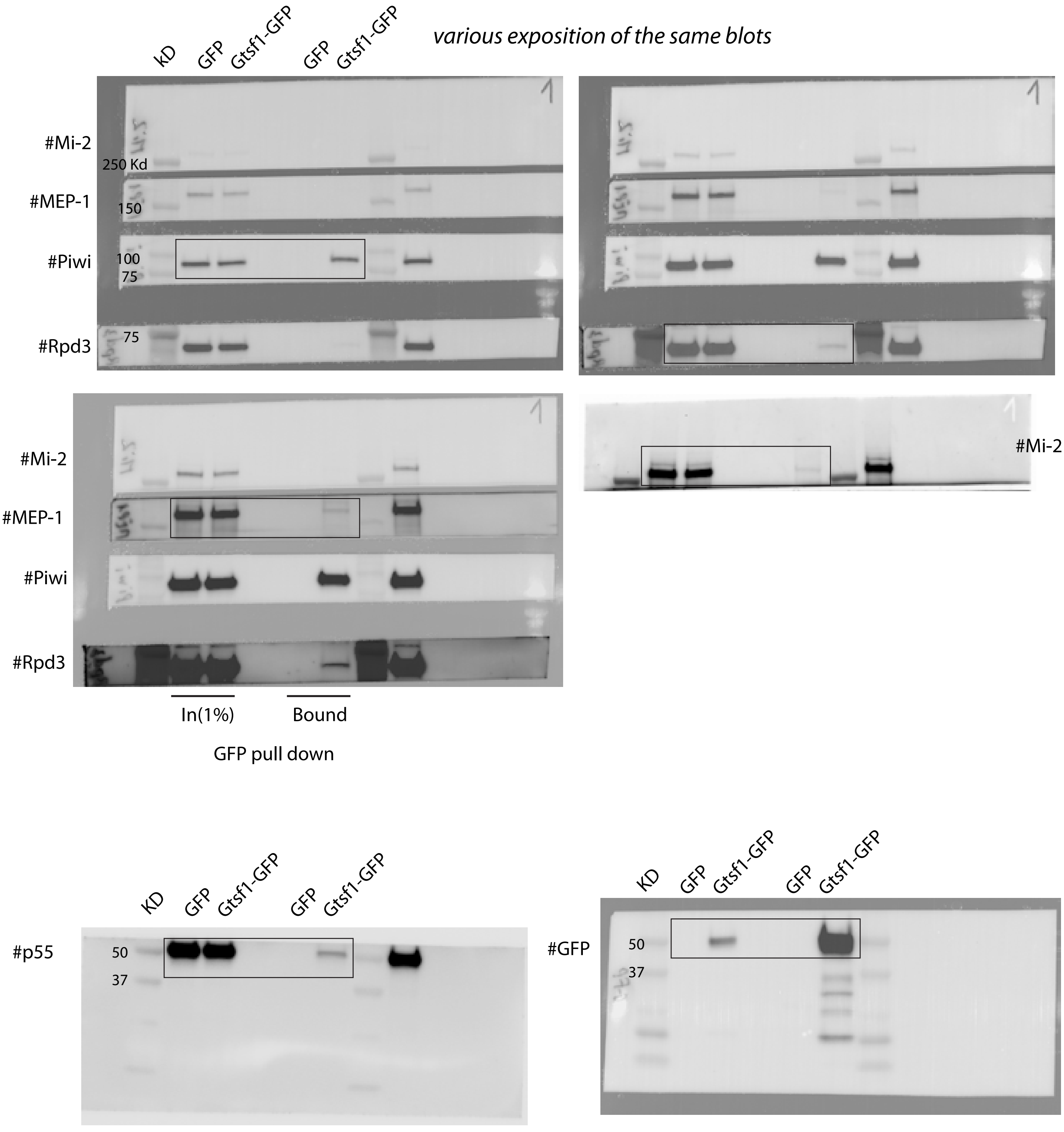

kD: precision Plus protein standards from BIO-RAD

Fig1d

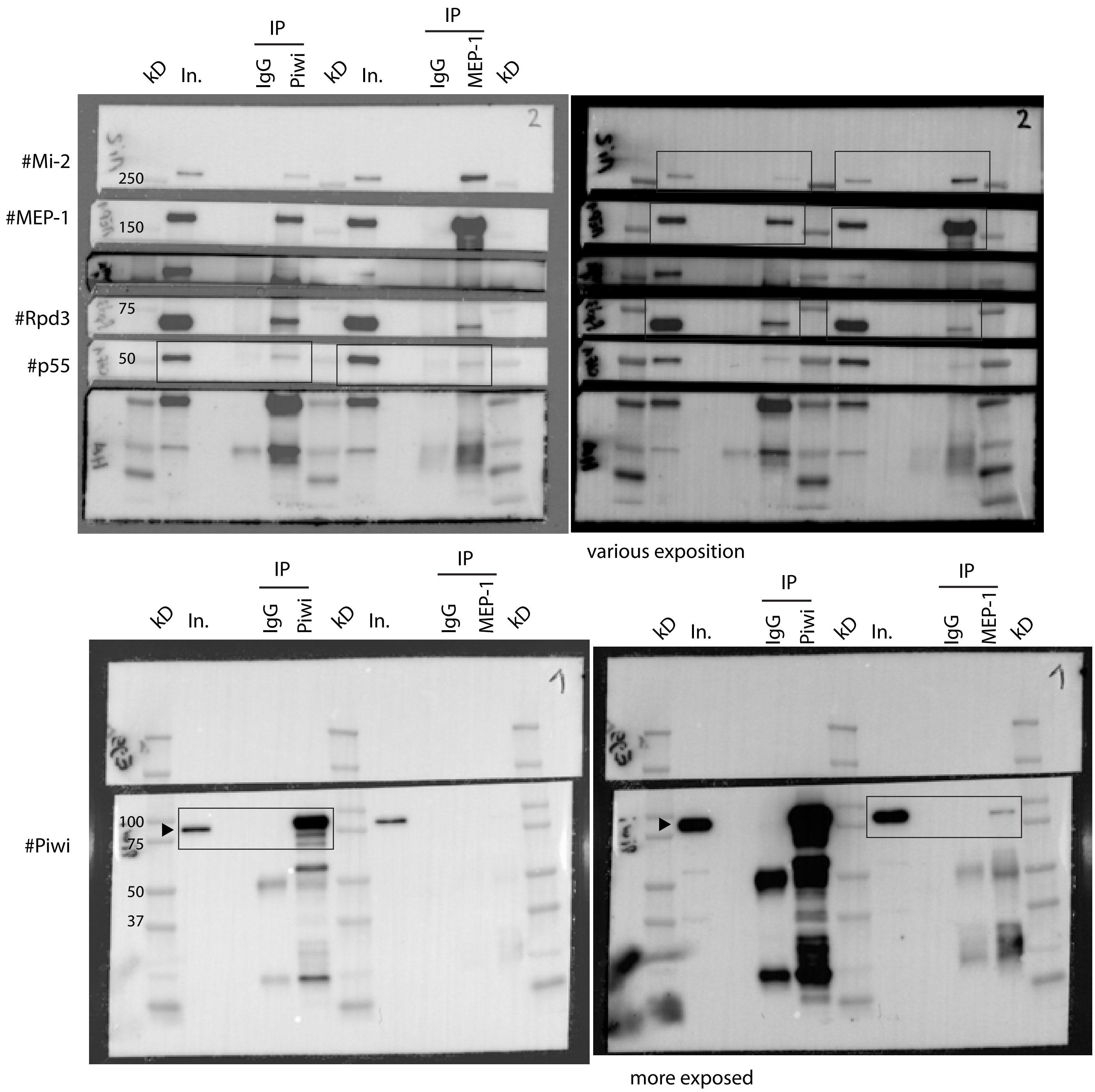

IP
IP
kD
In.
IgG
Piwi
kD
In.
IgG
MEP-1
kD
#Mi-2
#MEP-1
#Rpd3
#p55
250
150
75
50
various exposition
#Piwi
100
75
50
37
more exposed

Fig1d continued

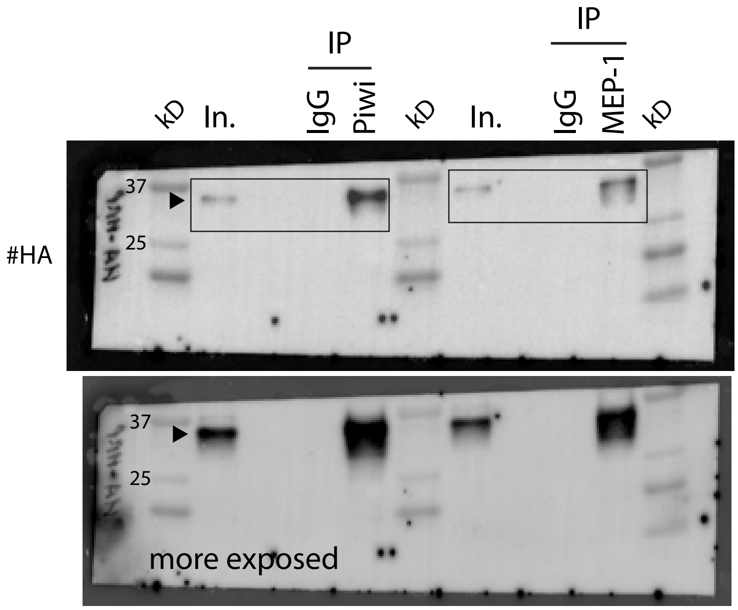

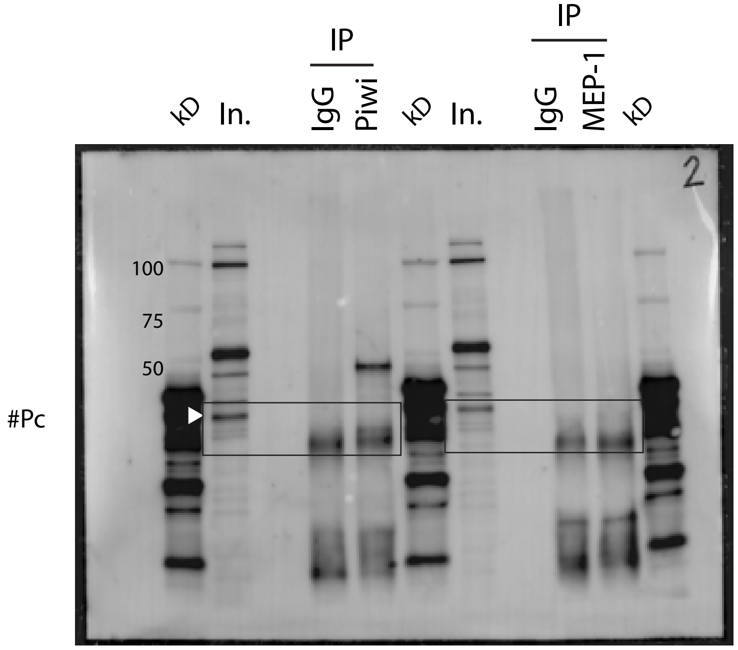

Fig2a

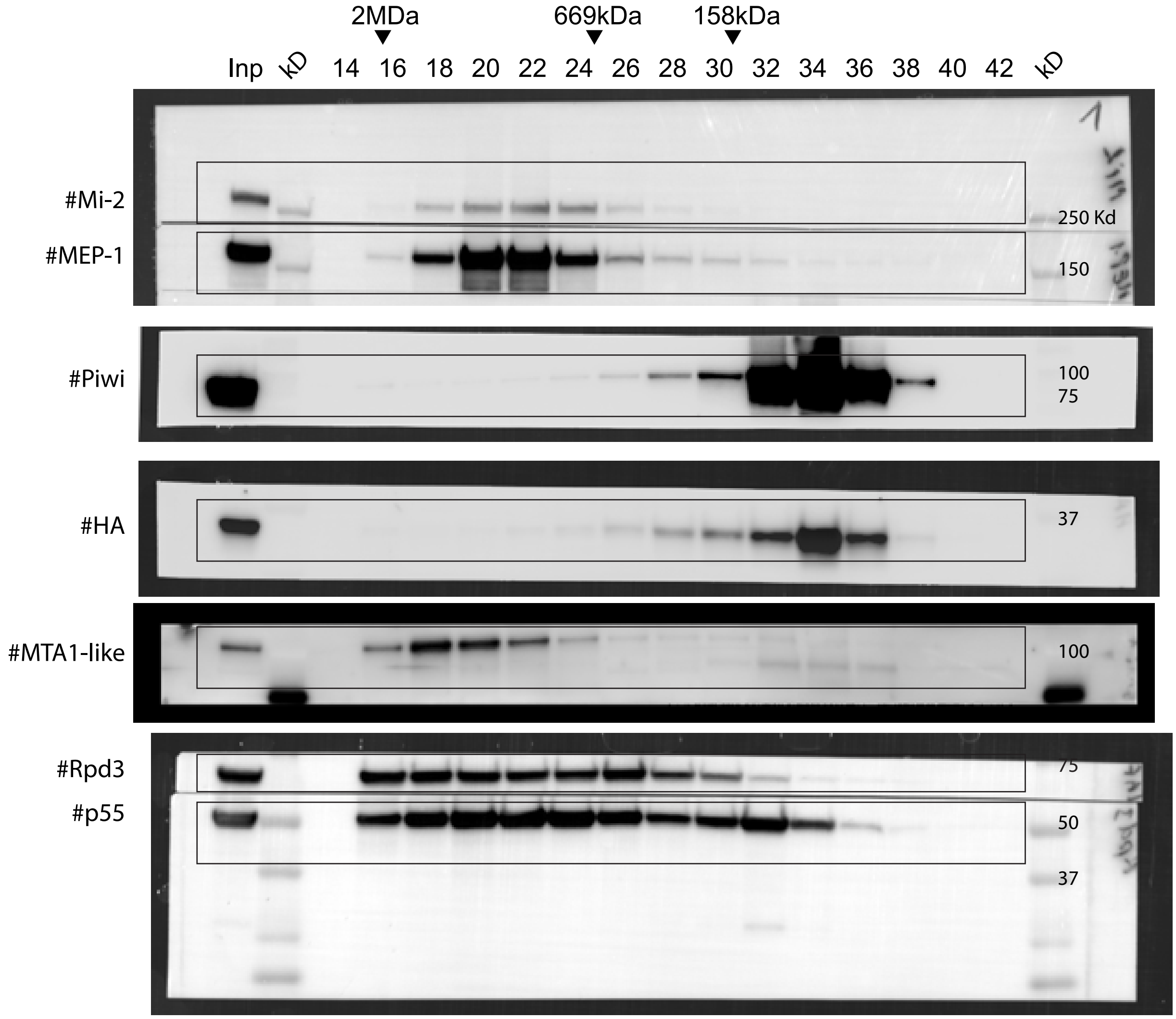
2MDa
669kDa
158kDa
Inp
kD
14 16 18 20 22 24 26 28 30 32 34 36 38 40 42
kD
#Mi-2
#MEP-1
250 Kd
150
#Piwi
100
75
#HA
37
#MTA1-like
100
#Rpd3
#p55
75
50
37

Fig2b

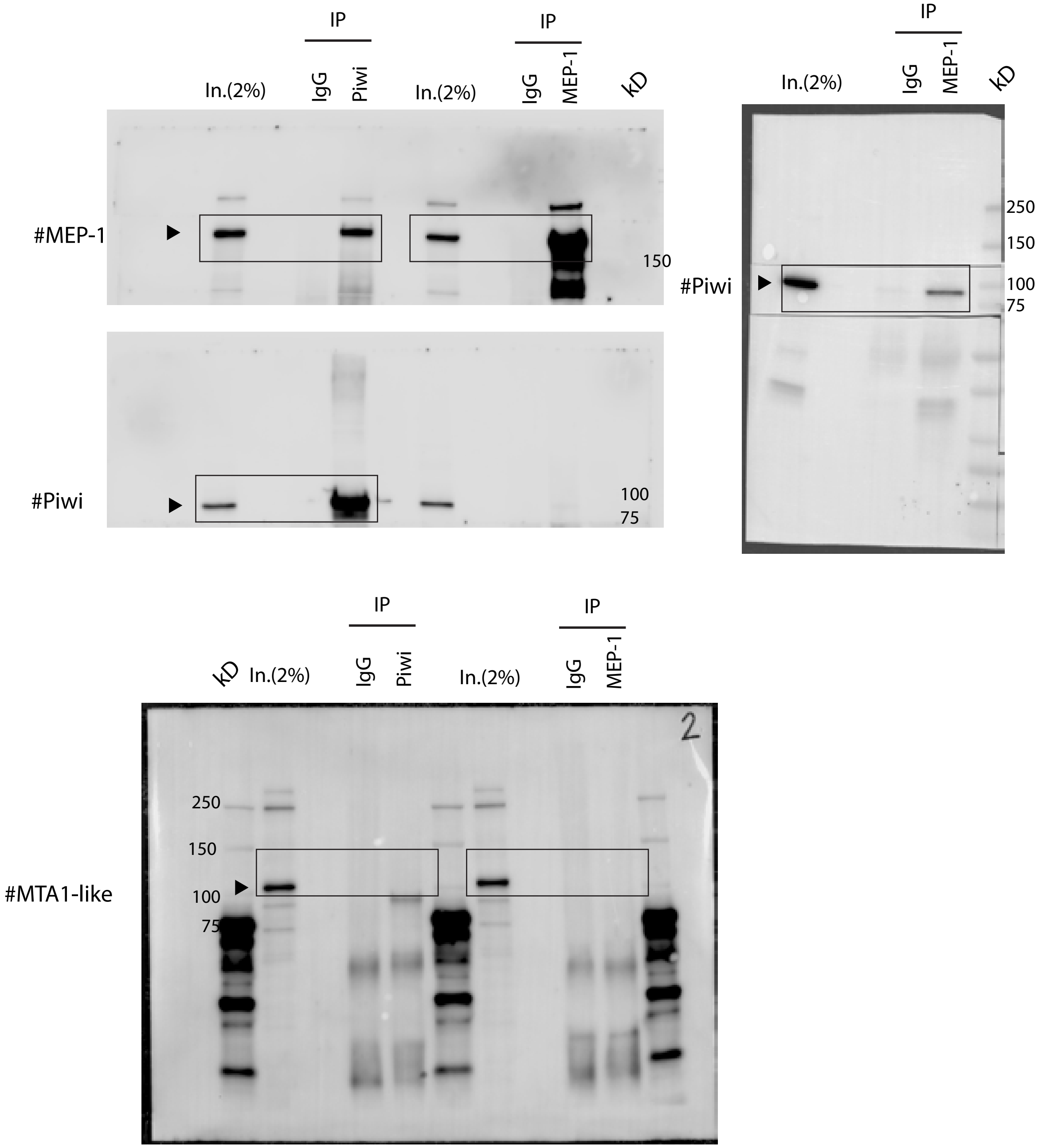
IP
In.(2%)
IgG
Piwi
In.(2%)
IgG
MEP-1
kD
#MEP-1
150
#Piwi
100
75
In.(2%)
IgG
MEP-1
kD
250
150
100
75
kD
In.(2%)
IgG
Piwi
In.(2%)
IgG
MEP-1
250
150
100
75
#MTA1-like
2

Figure 6a left panel

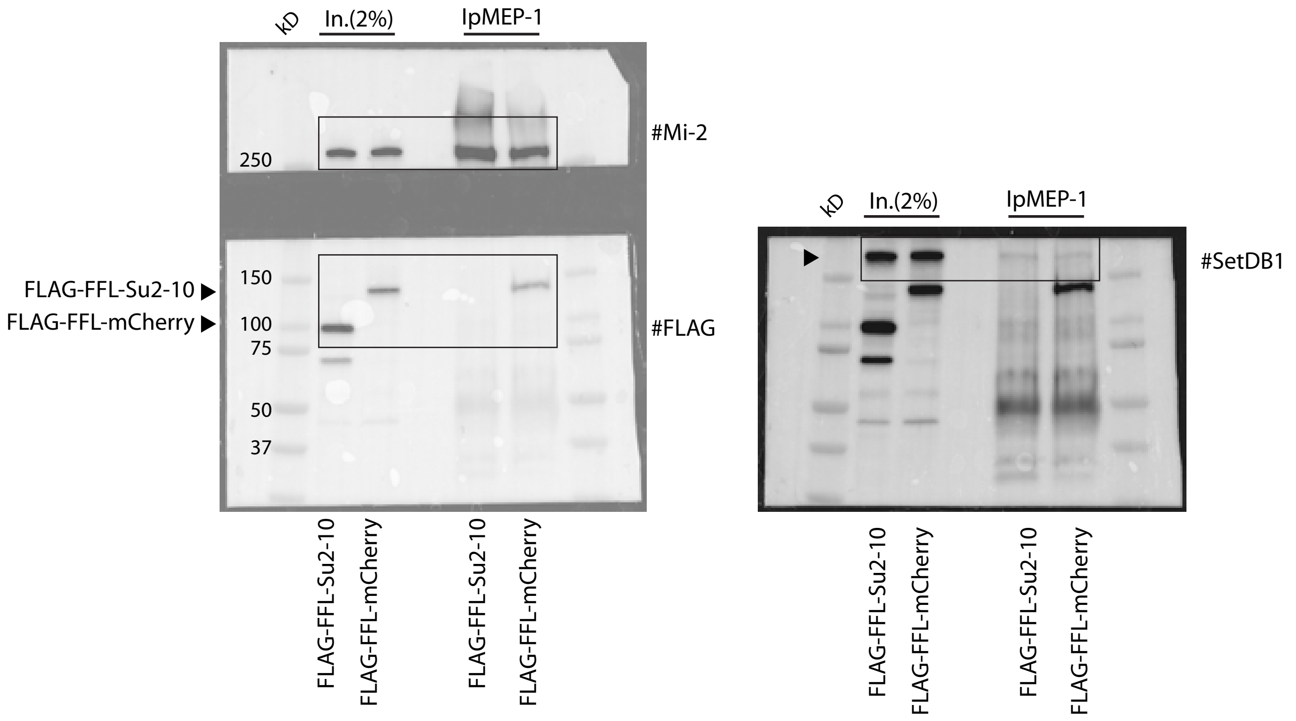

Figure 6a right panel

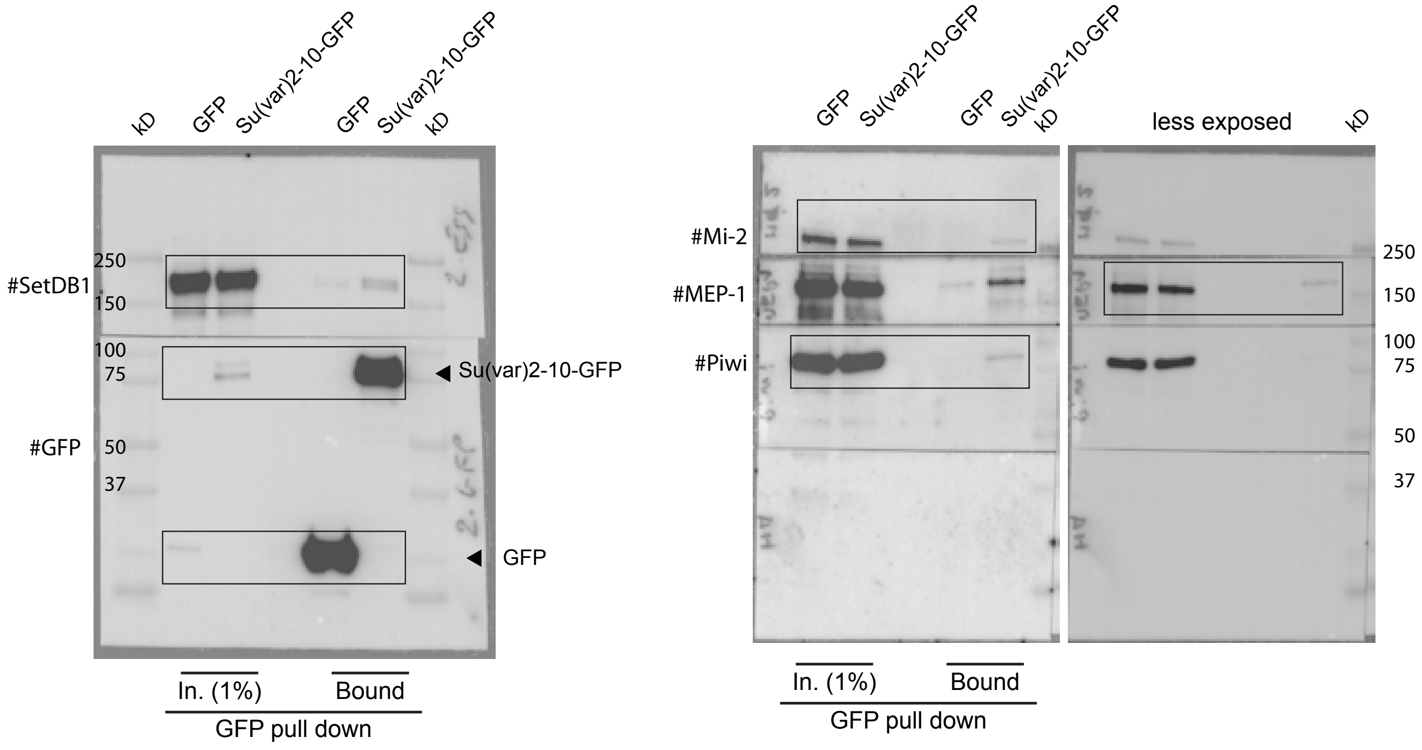

Supplementary Fig2b

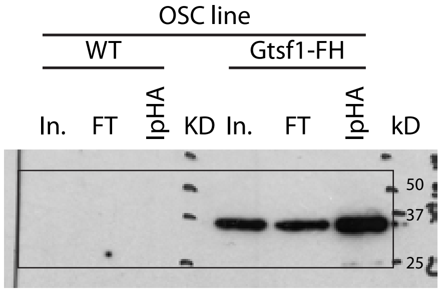

Supplementary Fig2c

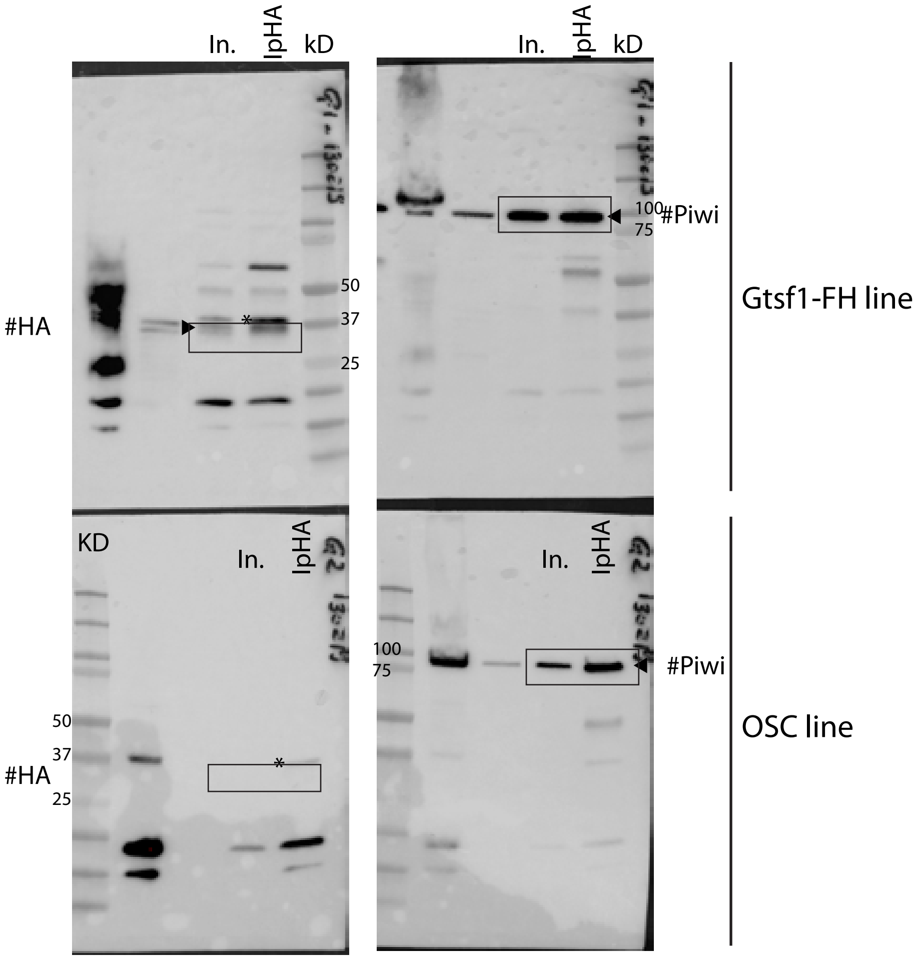

*: unspecific band

Supplementary Fig3a upper panel

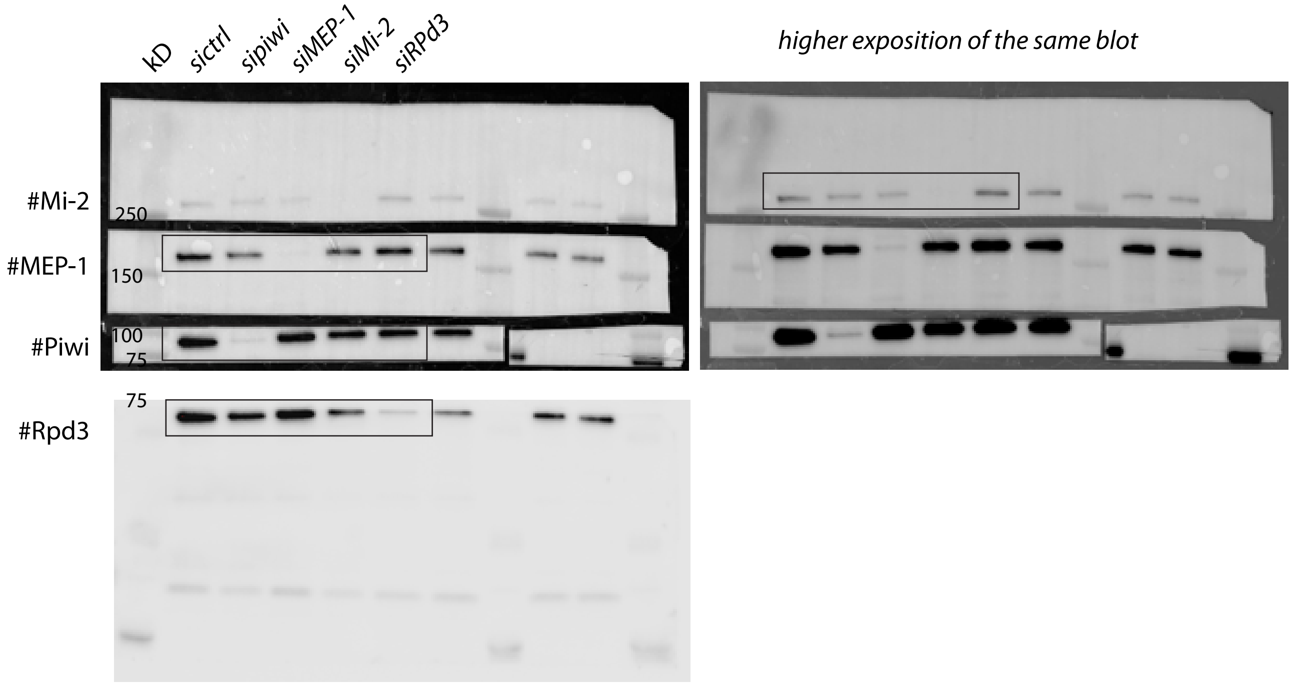

Supplementary Fig3a lower panel

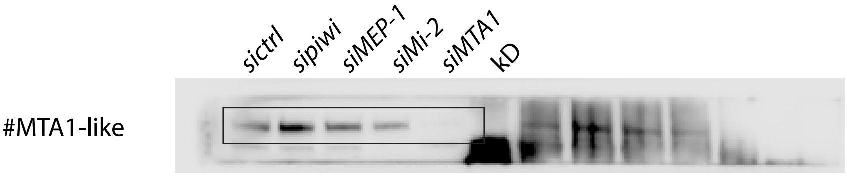

Supplement: Supplementary file 6 — Source Data [file 41467_2020_16635_MOESM6_ESM.zip › DATA SOURCE/Supplementary_Data_set_1.pdf]
